# Functions of primate amygdala neurons in economic decisions and social decision simulation

**DOI:** 10.1016/j.bbr.2021.113318

**Published:** 2021-07-09

**Authors:** Fabian Grabenhorst, Wolfram Schultz

**Affiliations:** Department of Physiology, Development & Neuroscience, University of Cambridge, Cambridge, CB2 3DY, UK

**Keywords:** Reward, Learning, Choice, Prediction, Social cognition

## Abstract

Long implicated in aversive processing, the amygdala is now recognized as a key component of the brain systems that process rewards. Beyond reward valuation, recent findings from single-neuron recordings in monkeys indicate that primate amygdala neurons also play an important role in decision-making. The reward value signals encoded by amygdala neurons constitute suitable inputs to economic decision processes by being sensitive to reward contingency, relative reward quantity and temporal reward structure. During reward-based decisions, individual amygdala neurons encode both the value inputs and corresponding choice outputs of economic decision processes. The presence of such value-to-choice transitions in single amygdala neurons, together with other well-defined signatures of decision computation, indicate that a decision mechanism may be implemented locally within the primate amygdala. During social observation, specific amygdala neurons spontaneously encode these decision signatures to predict the choices of social partners, suggesting neural simulation of the partner’s decision-making. The activity of these ‘simulation neurons’ could arise naturally from convergence between value neurons and social, self-other discriminating neurons. These findings identify single-neuron building blocks and computational architectures for decision-making and social behavior in the primate amygdala. An emerging understanding of the decision function of primate amygdala neurons can help identify potential vulnerabilities for amygdala dysfunction in human conditions afflicting social cognition and mental health.

## Highlights

• Amygdala neurons are sensitive to background reward (contingency).
• Amygdala neurons code principal decision variables: reward amount and timing.
• Amygdala neurons signal economic decision processes.
• Amygdala neurons predict the length of planned reward-acquiring sequences.
• Amygdala neurons simulate economic decisions of social partners.

## 1 Introduction

The amygdala has long been implicated in reward-guided behavior but a specific function for primate amygdala neurons in decision-making has only recently been demonstrated [[1], [2], [3], [4], [5], [6], [7], [8]]. Here we review data on the role of primate amygdala neurons in economic and social decision processes, and in reward valuation, which provides the necessary basis for decision-making. We suggest that consideration of the decision functions of primate amygdala neurons offers a new perspective on the amygdala’s broader role in linking sensory events and internal states to behavioral outputs, and on its involvement in human diseases.

The amygdala is a collection of nuclei located in the anterior part of the medial temporal lobe. It participates in a range of functions including emotion, learning, memory, attention and reward-guided behavior. Based on its anatomical connections, classical views considered the primate amygdala an interface between (i) hypothalamic and brainstem areas related to endocrine and autonomic functions, and (ii) limbic and neocortical regions related to cognitive functions [9]. The amygdala receives inputs from all sensory systems, including particularly rich visual inputs from the inferior temporal cortex, the orbitofrontal and medial prefrontal cortex, the hippocampus and rhinal cortices; it typically returns these projections, with additional outputs targeting the striatum, the hypothalamus, midbrain and brain stem [[10], [11], [12]]. These connections enable the amygdala to integrate information about sensory stimuli and internal states to regulate emotional, physiological and behavioral responses.

Early lesion studies suggested that amygdala damage in monkeys affects an animal’s ability to identify reinforcing stimuli [13]. Subsequently, studies in rodents established the amygdala as a critical brain system for fear conditioning and revealed the underlying cellular processes [14,15]. Human studies confirmed and elaborated the amygdala’s roles in emotion and reinforcement [[16], [17], [18]]. Initial neurophysiological investigations in monkeys described amygdala responses to visual and other sensory stimuli, some of which were related to the rewarding properties of the stimuli [[19], [20], [21]], and reward-related responses during instructed, multi-step behavioral schedules [22]. Subsequent studies demonstrated that amygdala neurons encode both the positive and negative value of visual stimuli during learning [[23], [24], [25]] and combine these value signals with the spatial position of stimuli [26] and other contextual information [[27], [28], [29]]. Lesion studies in monkeys and neuroimaging studies in humans also demonstrated amygdala involvement in reward processing [[30], [31], [32], [33],^44^].

In the present review, we focus on the neuronal signals mediating the primate amygdala’s more recently acknowledged functions in economic decision-making and in predicting others’ decisions in social contexts. We also consider key factors that influence the reward signals of amygdala neurons, which provide important inputs to decision processes.

## 2 Amygdala neurons are sensitive to background reward (contingency)

Primate amygdala neurons respond to conditioned, reward predicting stimuli and track the changing value of these stimuli during learning [25]. Accordingly, the question arises whether such responses would follow fundamental principles of animal learning theory. Standard concepts postulate that reward conditioning relies on the pairing between a reward and an arbitrary stimulus, as described by Pavlov. However, later assessments of conditioning showed that pairing is not the crucial aspect, as even repeated, well experienced pairing does not necessarily lead to behavioral conditioning or conditioned neuronal responses. Rather, what is required is specific information about the reward, which can be acquired when the reward depends, i.e. is contingent, on the stimulus. Such dependence occurs when more, or less, reward is given during the stimulus compared to its absence. Only in this situation does the stimulus carry specific reward information. Of course, reward occurrence results inadvertently in pairing with the stimulus; hence the notion of stimulus-reward pairing. By contrast, when a reward occurs both in absence of and during a stimulus, the stimulus is still well paired with the reward but, crucially, it carries no specific information about the reward—whether the stimulus occurs or not, the reward is the same. Carefully controlled experimental tests confirm this concept: the 'truly random' procedure demonstrates that non-contingent reinforcer occurrence does not lead to conditioning despite pairing [34]. A stimulus that is paired with a non-contingent reward does not become conditioned [35]. Thus, the crucial variable explaining learning is contingency, not the inadvertent pairing.

**Fig. 1:**
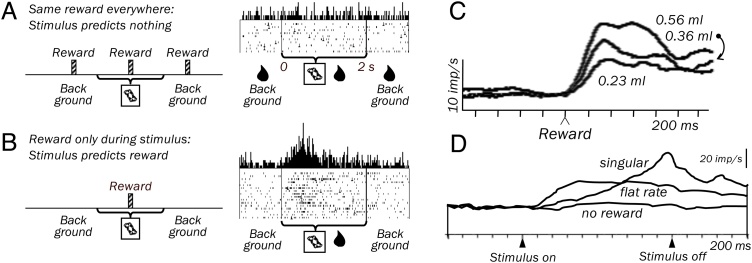
Signaling of fundamental reward parameters in amygdala: contingency, amount and timing. (A, B) Contingency: animal learning theory states that the important variable for acquiring reward prediction is contingency, not stimulus-reward pairing. (A) No response in single amygdala neuron to fractal stimulus when the same reward occurs with the same frequency also in the absence of the stimulus (‘background’). Despite being paired with the reward, this stimulus is not differentially informative about reward. (B) Response in same neuron to same stimulus with less background reward; only in this situation is the reward contingent on the stimulus; the stimulus predicts the reward. (C) Reward value coding in single amygdala neuron. Reward response increases monotonically with liquid amount. (D) Reward timing in population of 86 amygdala neurons. Different temporal profiles of reward expectation related activity reflect different instantaneous reward probabilities (indicated by three different stimuli; top, singular reward at stimulus end; middle, flat reward rate during stimulus, rewarded trials excluded from analysis; bottom, no reward).

These theoretical concepts demand to ask whether conditioned reward processing in amygdala neurons follows reward contingency rather than reward pairing. Lesions in the amygdala make rats insensitive to changes in background reward, indicating a general role in contingency-dependent learning [36]. To follow the 'truly random' procedure, a contingency test sets the reward probability independently during stimulus presence and absence (‘background’); it thereby controls for stimulus-reward pairing. We measured behavioral and neuronal responses in rhesus monkeys during such contingency tests [37]. Monkeys’ anticipatory licking behavior reveals conditioned responding when reward occurs less often during background compared to a visual stimulus, but similar licking, and thus no conditioned responding, when reward occurs with the same probability throughout both periods [37]. In such conditions, many amygdala neurons alter their stimulus response with changing contingency; they do not respond to visual stimuli when rewards occur as often during the stimulus as outside the stimulus period, even though the reward is paired with the stimulus (Fig. 1A). By contrast, the same neurons show substantial conditioned responses when the level of background reward drops below that during the stimulus (Fig. 1B). Only a smaller group of amygdala neurons responds to visual stimuli irrespective of contingency.

Thus, typical reward-conditioned responses in primate amygdala neurons reflect reward contingency rather than stimulus-reward pairing. Such acquired responses to reward-predicting stimuli would constitute useful inputs to neuronal mechanisms underlying informed economic decision-making. The specific mechanisms by which amygdala neurons process reward contingency from comparing stimulus-based reward and background reward remain to be uncovered.

## 3 Amygdala neurons encode reward amount

Given that amygdala neurons acquire responses to rewarding events [25] and that these responses reflect contingency [37], a participation in economic choice would also require graded coding of reward value. Reward amount is probably the most fundamental variable that determines reward value. Although rewards are valued on a subjective scale, the relationship between subjective and physical reward value is typically positive monotonic as long as satiety and other maximising factors are ruled out. Thus, subjective value follows physical reward amount in a ranked, ordinal fashion: more reward is usually better than less reward; measurable choices reveal that animals prefer larger to smaller reward amounts.

Reward amount scaling is reflected in the responses of primate amygdala neurons to reward receipt [38]: these responses increase or, in separate neurons, decrease monotonically with more reward (Fig. 1C). Importantly, choices are made relative to what is on offer, and decision makers tend to select the best reward within a given reward distribution. A reward that is the larger one of two rewards, and accordingly is chosen more frequently than its alternative, may become the smaller reward in comparison with a new, much larger reward; that same first reward will now be chosen less frequently than the large reward, even though it is physically unchanged. Thus, to be involved in economic decision-making, signals encoding reward amount in at least some amygdala neurons should vary systematically depending on the alternative reward on offer. This is indeed the case in many reward-sensitive amygdala neurons, both for responses to reward itself and for responses to reward-predicting stimuli. Neuronal responses to the smallest reward remain smallest, and to the largest reward remain largest, irrespective of their physical amounts [38]. Responses to reward-predicting stimuli show the same phenomenon, being smaller when the predicted reward is the smaller one of two available rewards, but being larger when the same reward is the larger one in the currently available set [29]. All these amygdala responses reflect the rank order of the rewards rather than their physical amounts.

The amygdala neurons reviewed above faithfully identify by their response which reward is the better one in any changing distribution of rewards. This quantitative reward code could provide critical inputs to an economic decision process. An important open question is whether the same amygdala neurons that encode quantitative reward-magnitude information are also sensitive to reward probability. Processing of both reward probability and magnitude in individual amygdala neurons would provide a substrate for computing key statistical properties of reward distributions, including expected value and variance.

## 4 Amygdala neurons are sensitive to reward timing

The predicted time of future reward fundamentally affects behavior, as evidenced in the temporal difference model of reinforcement learning [39] and the ramping, time-dependent evolution of decision variables in drift-diffusion and race models of decision-making [40,41]. If amygdala neurons participated in these learning and decision processes, they should have access to information about reward timing. When distinct visual stimuli predict particular instantaneous reward rates, the preparatory licking of monkeys suggests that animals have specific temporal reward expectations [42]. For example, a monkey would lick regularly throughout out a visual stimulus presentation when reward occurred at random times during the stimulus but show more focused licking at stimulus end if reward occurred reliably at that time. A group of amygdala neurons show responses that reflect these expectations: when reward is delivered at a fixed time point at stimulus end, activity in these neurons ramps up gradually toward stimulus end; however, when delivery of the same reward amount is spread out equally over the whole stimulus, the neuronal activity shows a smaller, tonic increase (Fig. 1D). Amygdala neurons show also time sensitivity at the reward itself. One type of response increases with increasing instantaneous reward rate, suggesting a positive, confirmatory relationship to expected reward reception. An opposite type of response increases with decreasing instantaneous reward probability, possibly reflecting a surprise in reward occurrence or even positive reward prediction error, as also seen in other task contexts [23].

The forms of sensitivity to temporal reward structure described above would allow amygdala neurons to participate in timing processes underlying learning and decision-making. Recent studies, described below, uncovered time-sensitive amygdala signals in decision tasks that require planning ahead and tracking progress over extended periods [4,5]. However, the role of amygdala neurons in choices between temporally distinct rewards and credit-assignment based on temporal information remains to be studied.

## 5 Amygdala neurons signal economic decision processes

Neuronal decision processes involve the translation of graded, parametric value signals, reflecting the evidence for the decision, to signals predicting the animal’s categorical choice. Primate amygdala neurons seem well suited to contribute to such reward-based decision processes. As described above, their flexible, context-sensitive value signals provide a substrate for integrating relevant evidence to decision inputs. An explicit choice signal computed from such values could also be broadly distributed by the amygdala, given its diverse efferent connections. Furthermore, lesions studies demonstrated essential amygdala contributions to decision-related processes including reinforcer devaluation [43,44], stimulus-reward learning [6] and probabilistic reversal learning [45,46]. Notably, amygdala inactivation does not seem to cause deficits in object choice when reward values are stable [47], in contrast to inactivation of the orbitofrontal cortex [48]. However, absent behavioral deficits after inactivation do not, per se, preclude amygdala involvement in decisions, as the amygdala may process choices in parallel with other brain systems or specialize in specific types of decision-making. Although the amygdala’s role in decision-making was often seen as restricted to reward valuation, the recent data described next suggest that primate amygdala neurons do contribute directly to economic decision-making, by encoding both the value inputs and choice outputs of decision processes.

In a series of studies, we examined the activity of primate amygdala neurons in a sequential economic decision-making task [1,4,5]. In this ‘save-spend task’, monkeys make consecutive choices to ‘save’ (i.e. accumulate) liquid rewards for future trials until deciding to ‘spend’ (i.e. consume) the saved reward amount. A variable, cued ‘interest rate’ governs increases in saved reward amounts over consecutive save choices, with high interest rates leading to exponential reward growths. The monkeys’ choices reflect knowledge of this task structure: they produce longer sequences of save choices when interest rates are high. Importantly, the task allows the animals to plan their choices multiple trials in advance, to obtain specific reward amounts through saving sequences of defined lengths. Challenge trials involving choices between saved and fixed reward amounts confirm that the animals successfully track saved reward amounts and anticipate final rewards.

**Fig. 2:**
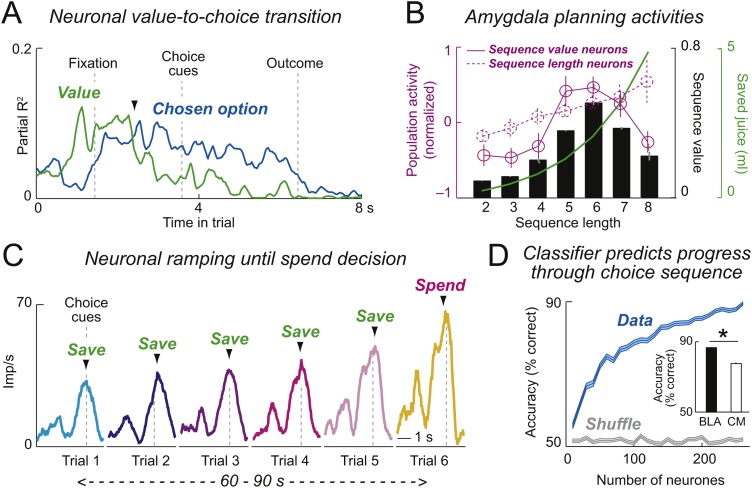
Amygdala responses during economic decisions. (A). Single amygdala neuron coding value input (green) and choice output (blue) of an economic decision process. Monkeys chose to save liquid reward for later or spend (consume) on current trial; they could plan save-spend choices (but not left-right actions) before choice cues. The neuron’s activity transitioned from signaling value to predicting the forthcoming save-spend choice (partial R^2^, sliding-window regression with value and choice regressors; arrowhead: onset of significant choice signal). (B). Amygdala neurons with planning activity for distant rewards. Neurons signaled the length of the planned choice sequence (dashed magenta curve, population activity) or its subjective value (solid magenta curve. Value activity was highest during sequences lasting six trials, which had the highest subjective value (black bars), i.e. were preferred by the animals, because they offered large reward (green curve) for moderate delay. (C). Single amygdala neuron tracking progress during sequential choices. Activity increased with consecutive save choices over 60-90 s until the monkey decided to spend the reward. (D). Decoding progress from amygdala neurons. Cross-validated progress-decoding accuracy of nearest-neighbor classifier. Inset: better decoding from basolateral than centromedial amygdala neurons.

During performance of the save-spend task, a significant number of amygdala neurons predict the monkeys’ save-spend choice on individual trials [1]. These choice-predictive activities (Fig. 2A, blue curve) are not explained by left-right actions, nor do they reflect subjective values of save-spend choices or chosen value, which are coded separately. Rather, amygdala neurons signal the animal’s abstract (action-independent) economic choice to spend reward for immediate consumption or save it for future trials. If these signals truly reflect the monkeys’ internal choice to save or spend, rather than simply encoding reward expectation, they should disappear when the requirement for an internal decision is removed. Indeed, the choice-predictive activities are often specific to freely made choices and disappear in a control task involving forced choices, confirming they do not reflect basic reward expectation. Some choice-predictive amygdala neurons show dynamic coding patterns within trials that comply with principles of computational decision models. In these neurons, subjective value signals related to specific save-spend choice options transition to explicit choice-predictive signals (Fig. 2A, green curve). The value-to-choice conversions of these neurons explicitly link the value inputs for decision-making to a choice output, consistent with theories of neural decision computation [49] and sensory decision signals in other brain areas [50].

More recent findings extend our beginning understanding of the amygdala’s decision functions. One study examined amygdala neurons in monkeys tracking the changing values of multiple visual stimuli and balancing exploration-exploitation decisions [7]. During this task, amygdala neurons signal both the value of choice options derived from a reinforcement-learning model and chosen option identity. In an economic choice task involving decisions between different juice rewards [8], amygdala neurons resemble those in orbitofrontal cortex [51] by encoding values of offered and chosen options, as well as the identity of the chosen juice. Finally, in the context of a social observational-learning task (described below), amygdala neurons signal both object values as decision inputs and the monkey’s trial-by-trial object choices [52]. Importantly, individual neurons encode explicit value-to-choice transitions and additional signatures indicative of decision computation, including sequential value comparisons and choice difficulty [52].

The data reviewed above suggest that primate amygdala neurons play a much more direct role in decision-making than traditionally thought. Specifically, the observation of explicit value-to-choice transitions in individual neurons [1] and other well-defined signatures of decision computation [52] raise the possibility of a decision computation performed locally within the amygdala’s neural circuits. Critical questions to resolve in future studies include whether the amygdala's decision signals are primarily referenced to visual choice objects or also extend to particular primary rewards and reward components, and whether some amygdala neurons encode choices in a purely abstract frame of reference, for example referenced to the animal's current view or focus of attention. In addition, it will be important to clarify whether amygdala decision signals contribute critically to value and choice coding in connected brain areas, as suggested by observations of diminished value coding in orbitofrontal cortex following amygdala lesions [2].

## 6 Amygdala neurons encode planned economic choice sequences

The best rewards are often distant, requiring careful planning and stepwise, sequential choices to reach internally set goals. In the save-spend task described above [1], amygdala neurons do not simply encode values and choices related to single trials but exhibit additional, sophisticated decision activities that are critical for optimal task performance [4,5]. One set of neurons shows prospective ‘planning activities’ that signal features of the animal’s internally planned choice sequence [4]. Specifically, neurons encoding ‘sequence value’ signal the subjective value of the current saving sequence (Fig. 2B, solid magenta curve), while a complementary set of neurons encodes the ‘sequence length’ by signalling the number of forthcoming save choices (Fig. 2B, dashed magenta curve). Sequence values are related to each animal’s preferences for specific choice sequences, reflecting both the benefits of specific rewards and costs due to delay and effort. Importantly, sequence value is a non-monotonic function of sequence length: depending on the current interest rate, sequence value is highest for intermediate sequence lengths (Fig. 2B, black bars) that offer a compromise between large rewards (Fig. 2B, green curve) and moderate delays. Notably, such planning activities often disappear during instructed reward-saving behavior, despite comparable reward timing and anticipation, and in control analyses are unrelated to reward proximity and expectation. These results and the distinct activities related to sequence value or sequence length suggest that amygdala planning activities do not simply reflect motivation or value. A recent study used functional magnetic resonance imaging (fMRI) in human volunteers to translate the save-spend task to human economic saving behavior [53] and identified corresponding signals in the human amygdala.

A distinct type of amygdala neuronal activity observed in the save-spend task explicitly signals the monkeys’ progress through a saving sequence [5]. Progress-tracking amygdala neurons show gradually increasing, ‘ramping’ activity over consecutive save choices until the animal decides to spend (Fig. 2C). Such responses occur in the absence of external progress cues and often specifically during internally guided choices. Importantly, the slope of amygdala ramping activities depends on the forthcoming sequence length, with steeper neuronal ramping for shorter sequences, suggesting adaptation of ramping activity to the monkey’s internal plan for a specific sequence length. Population decoding reveals a highly accurate neuronal progress code (Fig. 2D). Notably, neurons exhibiting planning signals and progress-tracking signals are more prevalent in the basolateral amygdala compared to centromedial amygdala. Basolateral neurons also encode progress more accurately in specific task periods (Fig. 2D, inset), suggesting particular importance for basolateral amygdala neurons in economic decisions.

Thus, the activity of amygdala neurons reflects the value and temporal structure of an animal's plan to obtain distant reward goals, and tracks the stepwise progress toward that goal. By specifying prospective reward goals, these amygdala signals could provide direction for behavioral plans encoded in frontal lobe areas [54,55]. This intriguing possibility remains a valuable question for future studies.

## 7 Amygdala neurons simulate decision processes of social partners

Primates do not only make choices individually but also observe the choices of their social partners. By observing their partners, primates can learn the values of objects to inform own decision-making and to predict their partners’ choices and intentions. The amygdala may play an important role in these social processes. Its neurons process social cues, such as faces [[56], [57], [58], [59]] and amygdala damage impairs social behavior [[60], [61], [62]]. The amygdala is also implicated in autism [59,63,64], which is marked by impoverished social cognition.

**Fig. 3:**
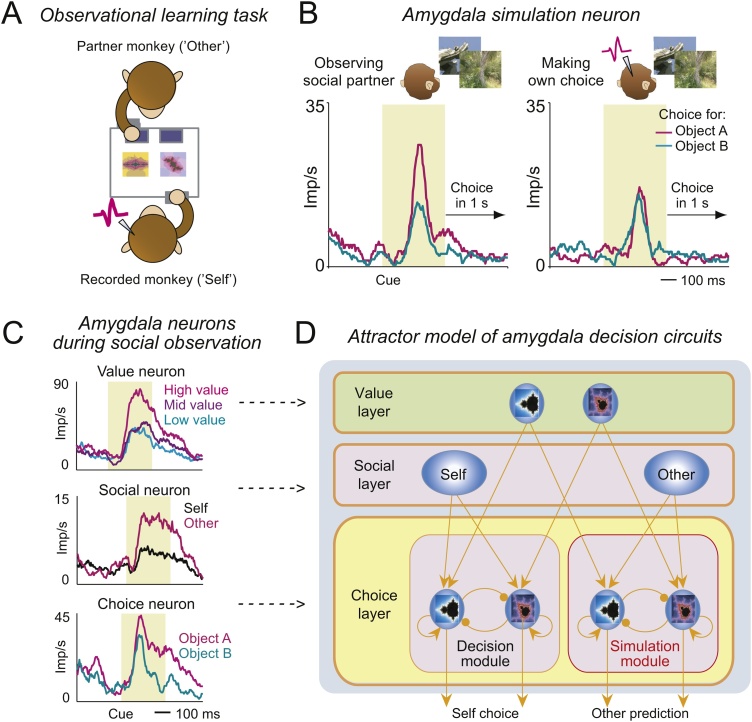
Amygdala responses during social decisions. (A). Two monkeys faced each other over a touch screen and took turns making choices between visual objects to learn object-reward probabilities (object values). (B) Amygdala simulation neuron. Neuron predicting object choice for the social partner monkey (left) but not for the recorded monkey (right). Neuronal responses were measured while the recorded monkey fixated the second object of two sequentially presented choice objects and before the partner monkey could move to choose the object on the touch screen. (C) Types of amygdala neurons recorded during the observational learning task. Object value neurons signal the value of specific choice objects, irrespective of whether value derives from own learning or social observation. Social neurons discriminate between self and other by showing differential activity on recorded monkey’s and partner’s trials. Different choice neurons signal either the recorded monkey’s own choices or the partner’s predicted choices (simulation neurons). (D) Biophysically plausible model of amygdala circuits for decision-making and social decision simulation, based on the recorded neuron types. Object-specific value neurons (Value layer) and self-other discriminating neurons (Social layer) provide convergent excitatory inputs to two separate decision systems (Choice layer) for computing own choices (Decision module) and for simulating social partner’s choices (Simulation module). Within each choice-layer module, groups of object-specific neurons endowed with recurrent excitatory connections compete with each other by mutual-inhibitory winner-take-all competition (mediated by inhibitory interneurons). Depending on activity in the social layer, value inputs selectively initiate competition in one of the two choice-layer modules.

A recent study examined the activity of amygdala neurons in a social situation in which two monkeys observe and learn from each other’s reward-based choices (Fig. 3A, [52]). When monkeys take turns choosing between visual objects, amygdala neurons signal object values learned from social observation and own experience in a common code. This neuronal object-value code is highly accurate and transferable between self and other, particularly in the lateral amygdala nucleus [52]. Such common, object-centric value coding could inform reward-learning irrespective of who is choosing and provides versatile inputs for own choices and social choice predictions. As observed previously [1], specific amygdala neurons convert these value signals to signals predicting the recorded monkey’s own choices. Remarkably, in a distinct group of amygdala neurons, the same choice-predictive activities occur immediately before the choice of the social partner monkey (Fig. 3B). Beyond choice predictions, these ‘simulation neurons’ encode three key signatures of a decision computation, including sequential value comparisons, sensitivity to decision difficulty and explicit value-to-choice conversion, suggesting a simulated decision process. In addition, specific amygdala neurons discriminate between self and other choice-trials.

The observed functional types of amygdala neurons (Fig. 3C) suggest a computational architecture in which common object-value signals provide inputs to two separate decision modules, computing the monkey’s own choices and simulating the choices of the social partner, respectively (Fig. 3D, [52]). This hypothesis was tested in a formal, biophysically plausible attractor neural-network model, based on an earlier non-social decision model [65]. In this model, both the decision module and simulation module contain choice-coding populations of neurons that are endowed with recurrent excitation, implemented by slow NMDA-receptor dynamics, and that compete with each other via mutual GABAergic interconnections (for details, see [52]). To perform a decision computation based on object-value inputs, each module requires additional, activating input from a separate social layer containing self-other discriminating neurons. For example, the simulation module would be engaged when a monkey anticipates the partner’s choice through convergence of object-value signals and other-specific signals onto decision neurons. The network reproduces some of the key activity patterns of the recorded amygdala neurons, including the selective decision signals of simulation neurons only on partner’s but not own trials and the dependence of the simulated decision signal on the partner’s choice difficulty. Further, individual amygdala neurons jointly encode values and self-other discriminating signals before they encode choices, matching the information flow implied by the model. The data and computational model suggest a neurobiological account of social cognition as neural decision computation: By performing decision computations during social observation, amygdala simulation neurons may allow primates to reconstruct their social partners’ mental states.

The amygdala is unlikely to perform these sophisticated operations in isolation. The anterior cingulate cortex contains neurons that predict partner’s choices during competitive interactions [66], neurons in both amygdala and anterior cingulate cortex participate in prosocial decision-making [3] and processing social gaze [67,68], and both areas coordinate their activity during prosocial choices [69]. Notably, a recent study reported that amygdala neurons encode the social rank of conspecifics, cued by pictures of monkeys’ faces, in the same manner as they encode the reward value of non-social conditioned stimuli; anterior cingulate and orbitofrontal cortex lacked such coding of social hierarchical rank [58].

Thus, recent data suggest important contributions of primate amygdala neurons in social decision-making and predicting others’ choices. These data may help explain the effects of amygdala lesions on social behaviour [60,61], the involvement of amygdala in atypical social cognition [59,63,64], and relationships of amygdala structure and function to primates’ social status [70]. Accordingly, it will be important to investigate whether amygdala neurons participate in even more sophisticated social functions, such as processing rewards from the perspective of a partner even when the partner's subjective valuation differs from one's own.

## 8 Conclusions

The findings reviewed above suggest an emerging account of how amygdala neurons and circuits contribute to individual decision-making and social decision simulation. A role for the primate amygdala in implementing a decision mechanism is consistent with observed value-to-choice conversions in individual amygdala neurons [1], with additional signatures of decision computation encoded by amygdala neurons [52], and with features of amygdala inhibitory microcircuits [71,72] that could implement decision computations by mutual-inhibitory winner-take-all competition. Although amygdala inactivation does not necessarily cause choice deficits when reward values are stable [47], the amygdala makes essential contributions to decision-making in situations requiring ongoing valuation [6,31].

How do amygdala decision signals relate to decision signals found in other brain structures? The amygdala and orbitofrontal cortex receive prominent sensory inputs from all modalities [12], which predisposes both structures to process decisions about typically multisensory rewards. This view is supported by neurophysiological studies that recorded from both structures in the same task and identified largely similar types of signals [8,51]. Moreover, lesion studies emphasize the importance of amygdala-orbitofrontal interactions in decisions requiring value updating [73]. Given its prominent reward functions, it seems unlikely that the amygdala would participate in basic perceptual decision processes. Nevertheless, amygdala decision neurons recapitulate features of perceptual decision processes observed in random-dot motion and vibrotactile decision tasks [50,74], including the dynamic translation from graded evidence to binary choice by single neurons, the dependence of decision signals on evidence strength, and the tuning of decision neurons to specific choice options [1,52]. As described above, amygdala neurons are more strongly involved in processing social hierarchy information than neurons in orbitofrontal cortex and anterior cingulate cortex [58]. This finding could suggest a relative specialization of the amygdala for social processes, compared to other structures, which may extend to social decision-making.

The reviewed parallel neurophysiological studies on amygdala decision and social functions also inform the broader question of whether the amygdala is specialized for social behavior, as suggested by the Social Brain Hypothesis [75], or whether its social functions can be explained by simpler processes, including stimulus-reinforcer learning [76] and decision computation. Notably, amygdala simulation neurons [52] encode the same decision process during social observation as encoded by separate neurons during own decision-making. This finding suggests that a complex social function such as predicting others’ choices may be supported by an essentially non-social neural decision computation, albeit in dedicated “social neurons”. The self-other discriminating neurons observed in the same study may constitute a critical building block for setting up social representations, as suggested by computational modelling [52]. A similar principle seems to underlie the evaluation of social hierarchy and gaze performed by amygdala reward neurons [58,68].

The decision signals and planning activities in amygdala neurons described above may inform our understanding of amygdala dysfunction in human psychiatric conditions, including mood disorders that impact on the motivation to make decisions and pursue future rewards [77]. The amygdala is also implicated in autism and other conditions with atypical social cognition, including social anxiety [59,64]. The data reviewed above and our model of amygdala circuits for social decision simulation [52] may offer new insights into these conditions by specifying single-neuron building blocks and computational architectures for social cognition, and thereby identify potential vulnerabilities for dysfunction. For example, instability in the networks’ attractor dynamics or a lack of ‘social’ self-other neurons could result in absence of dysfunction of the network’s simulation neurons, which could impair an individual’s ability to predict the intentions and decisions of social partners. Conversely, over-activity of the simulation module could result in exaggerated neuronal simulation of other’s decision processes, which could provoke somatic symptoms typical of social anxiety via the amygdala’s outputs to physiological effector systems [12].

## Declaration of competing interest

None.
